# Exploration of the core protein network under endometriosis symptomatology using a computational approach

**DOI:** 10.3389/fendo.2022.869053

**Published:** 2022-09-02

**Authors:** Fatima El Idrissi, Mathilde Fruchart, Karim Belarbi, Antoine Lamer, Emilie Dubois-Deruy, Mohamed Lemdani, Assi L. N’Guessan, Benjamin C. Guinhouya, Djamel Zitouni

**Affiliations:** ^1^ Univ. Lille, UFR 3S, Faculté Ingénierie et Management de la Santé, Lille, France; ^2^ Univ. Lille, UFR 3S, Faculté de Pharmacie, Lille, France; ^3^ Univ. Lille, CHU Lille, ULR 2694 - METRICS, Lille, France; ^4^ Univ. Lille, Inserm, CHU-Lille, Lille Neuroscience & Cognition, Lille, France; ^5^ Univ. Lille, Inserm, CHU Lille, Institut Pasteur de Lille, U1167 - RID-AGE - Facteurs de risque et déterminants moléculaires des maladies liées au vieillissement, Lille, France; ^6^ Univ. Lille, UMR CNRS 8524, Laboratoire Paul Painlevé, Villeneuve d’Ascq, Cedex, France

**Keywords:** endometrium, cell signaling, female infertility, systems biology, text-mining

## Abstract

**Background:**

Endometriosis is defined by implantation and invasive growth of endometrial tissue in extra-uterine locations causing heterogeneous symptoms, and a unique clinical picture for each patient. Understanding the complex biological mechanisms underlying these symptoms and the protein networks involved may be useful for early diagnosis and identification of pharmacological targets.

**Methods:**

In the present study, we combined three approaches (i) a text-mining analysis to perform a systematic search of proteins over existing literature, (ii) a functional enrichment analysis to identify the biological pathways in which proteins are most involved, and (iii) a protein–protein interaction (PPI) network to identify which proteins modulate the most strongly the symptomatology of endometriosis.

**Results:**

Two hundred seventy-eight proteins associated with endometriosis symptomatology in the scientific literature were extracted. Thirty-five proteins were selected according to degree and betweenness scores criteria. The most enriched biological pathways associated with these symptoms were (i) Interleukin-4 and Interleukin-13 signaling (p = 1.11 x 10^-16^), (ii) Signaling by Interleukins (p = 1.11 x 10^-16^), (iii) Cytokine signaling in Immune system (p = 1.11 x 10^-16^), and (iv) Interleukin-10 signaling (p = 5.66 x 10^-15^).

**Conclusion:**

Our study identified some key proteins with the ability to modulate endometriosis symptomatology. Our findings indicate that both pro- and anti-inflammatory biological pathways may play important roles in the symptomatology of endometriosis. This approach represents a genuine systemic method that may complement traditional experimental studies. The current data can be used to identify promising biomarkers for early diagnosis and potential therapeutic targets.

## Introduction

Endometriosis is a gynecological inflammatory disease affecting women of reproductive age (1, 2). Approximately 200 million women worldwide, 10% to 15% of women of reproductive age and 2.5% of postmenopausal women are affected by endometriosis (3, 4). Three main forms of endometriosis are described: (i) ovarian endometriosis, (ii) superficial peritoneal endometriosis and (iii) deep infiltrating endometriosis (DIE) (1, 5), the latter being recognized as the most severe form (6, 7). Endometriosis is defined by implantation and invasive growth of endometrial tissue in extra-uterine locations, causing chronic pelvic pain, dyspareunia, dysmenorrhea, menorrhagia, bowel symptoms, and infertility (1, 6, 8). Endometriosis symptoms are associated with substantial reductions in quality of life (9, 10). Living with severe cyclic or continuous pelvic pain can lead to stress, anxiety, depression and absenteeism from work (11, 12).

Endometriosis is a multifactorial disease, with complex pathophysiological mechanisms, of which genetic and environmental components are still poorly evaluated (13, 14). The etiology of endometriosis is not completely understood. Several hypotheses have been put forward concerning the histological origins of endometriosis; the most accepted theory being Sampson’s theory of retrograde menstruation, which involved fragments of menstrual endometrium being disseminated through the fallopian tubes (7, 9, 13). However, this phenomenon is observed in nearly 90% of women, suggesting that immune and hormonal dysfunctions may add to the observed fragmentation, yielding the adhesion, survival and proliferation of the lesions (1, 2, 10). Several mechanisms such as exacerbated production of growth and pro-inflammatory factors, an increase in estradiol expression combined with progesterone resistance, and an overexpression of reactive oxygen species might be involved in the development of endometriosis (10, 14, 15). Distinct immunological abnormalities involving angiogenesis, vasculogenesis and inflammation have been well described. These processes involve molecules including the *VEGF* factor that triggers angiogenesis, *tumor necrosis factor* (*TNF*)-α, which plays an essential role in increasing proliferative potential and acts primarily as a precursor to initiating an inflammatory response by activating a cascade of other cytokines, such as *IL-1, IL-6* and *CXCL8* (8, 16–18). The ineffectiveness of using anti-inflammatory agents to treat endometriosis shows that the disease is more related to the loss of balance between pro- and anti-inflammatory molecules (4). It appears clearly today that to understand the complexity of this disease, it is necessary to study the processes involved as a whole, as well as potential interactions between their components.

There is currently no treatment for endometriosis. On the other hand, the time between the development of the first lesions and the diagnosis is estimated between 7 and 10 years (2, 10). Thus, two major challenges must be met: identification of early diagnostic biomarkers, and that of potential therapeutic targets. An increasing number of studies are based on the search for biomarkers involved in endometriosis in order to develop less invasive diagnostic methods (i.e., urine tests, blood tests) (19, 20). The biological complexity under endometriosis has been previously addressed using computational biology approaches on the basis endometriosis-related alternations (e.g. immune cell infiltration) (21) or the development and progression of endometriosis (22). However, to the best of our knowledge, no study had used endometriosis-related symptoms to build up its underlying biomolecular processes.

Finding useful information in the genetic data generated for endometriosis is very challenging and computational biology can be helpful. Our study used tools of computational biology to identify biological processes and protein networks underlying the symptomatology of endometriosis. We combined text-mining, functional enrichment and protein-protein interaction analyses to suggest some biomarkers or therapeutic targets deserving further exploration.

## Materials and methods

### Protein collection with text-mining

The Medline database was used as a data source to perform a systematic search of genes associated with endometriosis symptomatology. The PubMed^®^ search query [“endometriosis” AND (“dysmenorrhea” OR “metrorrhagia” OR “dyspareunia” OR “dyschesia” OR “symptoms”)] was used to retrieve the PMIDs related both to endometriosis and at least one of its symptoms. Articles, from the inception of PubMed until December 2020, which considered a human model, dealt with of reproductive age (i.e. women between 13-44 years old), and published in English, were included. A text-mining of genes related to all types of endometriosis was carried out using the Pubtator resource, which has been developed as an extension of the NCBI, to provide access to biomedical and genomic information (https://www.ncbi.nlm.nih.gov/research/pubtator/) (23, 24). Then, the genes identified with Pubtator in the title, abstract and full text of the PMIDs list was retrieved using the panda library in Python language (www.python.org/).

Text-mining was performed using the GNormPlus Pipeline, which includes two modules: gene mention recognition and gene name normalization. This pipeline has an accuracy of 87.1% (25). We then used 309 UniProtKB Retrieve/ID mapper (https://www.uniprot.org/) to retrieve the UniProtKB protein identifiers associated to these Gene ID (26, 27). Uniprot provides a comprehensive collection of all known, manually annotated protein sequence data.

### Gene set enrichment

The essential part of this analysis consisted in translating the genetic signatures into information that can help to understand the underlying biological mechanisms. The annotations determine which proteins are significantly enriched in an entry list compared to a reference list. Gene Ontology (GO) enrichment of the collected proteins was first performed using the GeneCodis (https://genecodis.genyo.es/), with annotation from GO Cellular Component, GO Molecular Function and GO Biological Process categories (28, 29). The most enriched annotations were then visualized using the ggplot2 package in R language (www.r-project.org). Functional enrichment analysis of the proteins was subsequently performed and visualized using the Reactome Pathway Database (https://reactome.org) (30, 31). The functional pathways were sorted in ascending order according to their p-value, and proteins involved in the 10 most significantly enriched functional pathways (i.e. with the lowest p-values) were selected for subsequent analysis.

### Protein-protein functional interaction

The STRING protein query database was used to build a protein-protein functional interaction network in Cytoscape 3.7.2 (32). STRING is known as the primary source to depict and visualize the interaction among various proteins (32). The minimum combined score was set at 0.9 to retain only highest-confidence functional and physical interactions. In the network the nodes correspond to proteins and the edges to the interactions between each protein. We then used the CentiScaPe Cytoscape plug-in to calculate the node degree and betweenness centrality of each protein. The nodes (proteins) that had a degree centrality and a betweenness centrality greater than or equal to the mean were identified as key proteins more likely to modulate symptoms of endometriosis.

## Results

### Protein collection

**Figure 1:**
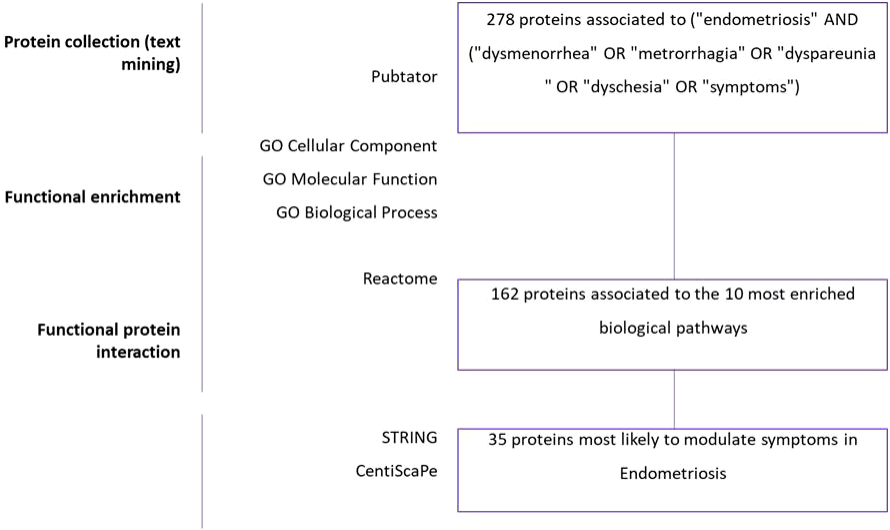
Summary of data mining results. Text-mining: Three hundred and nine genes were found by using Pubtator and total of 278 proteins ID were reviewed on Uniprot. Gene Ontology: Biological process, Cellular component, Molecular function analyses were performed in GeneCodis. Gene set enrichment: Pathway analysis was performed in GeneCodis to enrich 278 genes. Then, 162 significant genes were derived by protein-protein interaction analysis using STRING and Cytoscape. Thirty-five significant genes were selected for the final analysis with degree and betweenness criteria using Centiscape and Cytoscape.

The workflow of the study is described in **Figure 1**. The number of articles published on endometriosis symptomatology has been growing exponentially in recent years (**Supplementary Figure 1**). Our PubMed database queries yielded 2,177 articles published on the topic from 1990 to 2020. The PMIDs of these articles were downloaded and processed using Pubtator. A total of 309 genes were initially obtained, and then converted into unique protein identifiers, which were translated into 278 reviewed proteins, which in turn were linked for further analysis.

### Enrichment analysis

**Figure 2:**
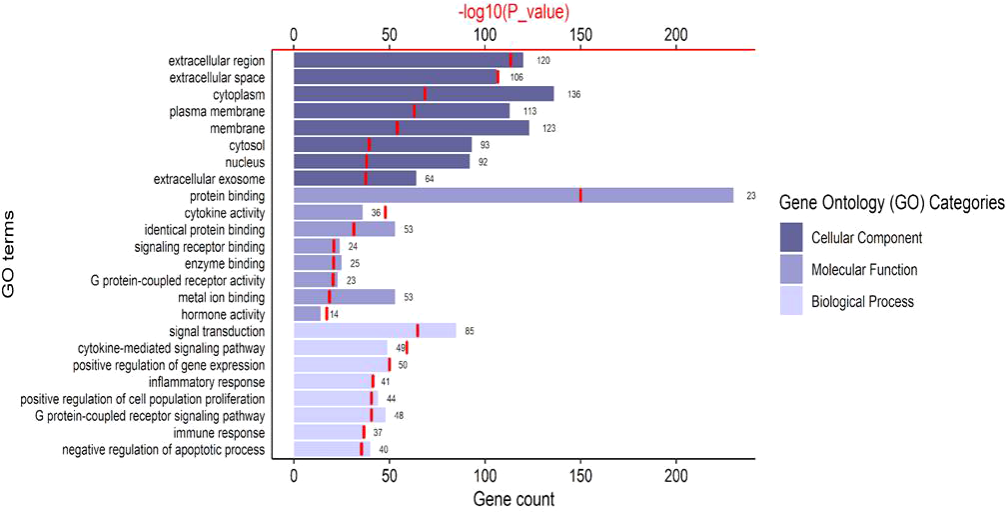
The top 8 significant Gene Ontology terms of common genes. The bar charts represent the counts of genes classified in the Cellular Components, Molecular Functions, Biological Pathways, respectively. The red line chart represents the significance of enrichment terms (-log10(p_value)).

The top eight enriched terms of the Cellular component, Molecular Function and Biological Process are presented in **Figure 2**. Cellular Components showed an enrichment of the proteins expressed in *extracellular region, extracellular space, cytoplasm, plasma membrane, membrane, cytosol, nucleus* and *extracellular exosome*. Molecular Function annotations showed that the proteins involved in the top eight terms were expressed in binding proteins (including *protein binding, identical protein binding, signaling receptor binding, enzyme binding, metal ion binding*), *cytokine activity, G protein-coupled receptor activity* and *hormone activity*. Biological Process annotations revealed that the most highly enriched terms were *signal transduction, cytokine-mediated signaling pathway, positive regulation of gene expression, inflammatory response, positive regulation of cell population proliferation, G protein-coupled receptor signaling pathway, immune response* and *negative regulation of apoptotic process*. The two most enriched Biological Process terms were signal transduction (p = 7.40 x 10^-62^) and cytokine-mediated signaling pathway (p = 1.33 x 10^-56^), which were closely related to the pathology of endometriosis.

**Figure 3:**
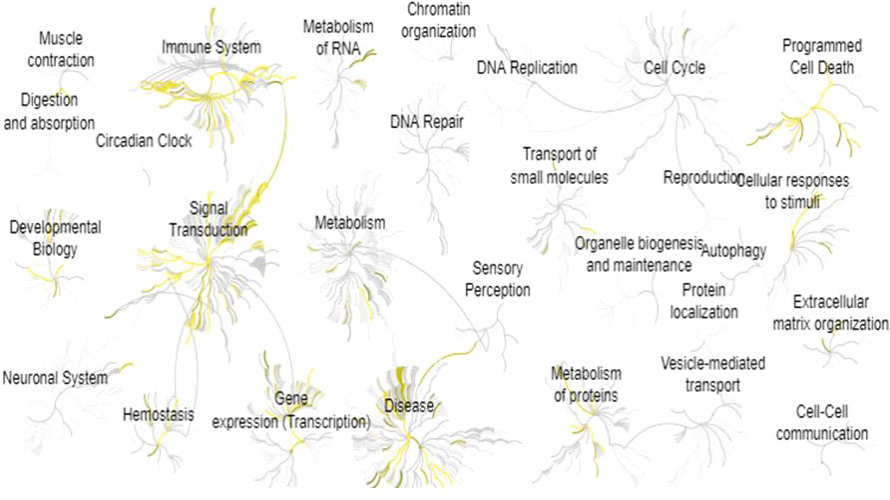
Pathway enrichment analysis for all the 278 proteins identified by text-mining. This analysis was performed by using Reactome Pathway Database. Yellow means pathways that are significantly overrepresented.

All the proteins mined were analyzed in the Reactome Database in order to visualize biological processes associated with endometriosis’ symptomatology. The 28 global pathways analyses are shown in **Figure 3**. The 10 most enriched pathways were selected: (1) Interleukin-4 and Interleukin-13 signaling (p = 1.11 x 10^-16^), (2) signaling by Interleukins (p = 1.11 x 10^-16^), (3) Cytokine signaling in Immune system (p = 1.11 x 10^-16^), (4) Interleukin-10 signaling (p = 5.66 x 10^-15^), (5) Signal transduction (p = 6.93 x 10^-12^), (6) Extra-nuclear estrogen signaling (p = 3.37 x 10^-9^), (7) Signaling by GPCR (p = 4.34 x 10^-9^), (8) GPCR ligand binding (p = 4.43 x 10^-9^), (9) Immune System (p = 4.56 x 10^-9^), (10) Interleukin-1 processing (p = 1.50 x 10^-7^) (**Table 1**). We extracted all the proteins involved in the 10 biological pathways mentioned above and removed the duplicates. One hundred sixty-two unique proteins from the 10 most enriched pathways were retained for protein-protein interaction analysis.

**Table 1:** Summary of the 10 most enriched biological pathways, grouping 162 unique proteins associated to endometriosis symptomatology using Reactome Pathway Database.

Pathway name	Count	Total genes in genome	Entities p-value
**Interleukin-4 and Interleukin-13 signaling**	30	111	1.11 x10^-16^
**Signaling by Interleukins**	59	457	1.11 x10^-16^
**Cytokine signaling in Immune system**	70	804	1.11 x10^-16^
**Interleukin-10 signaling**	17	45	5.66 x10^-15^
**Signal Transduction**	117	2574	6.93 x10^-12^
**Extra-nuclear estrogen signaling**	15	80	3.37 x10^-9^
**Signaling by GPCR**	46	706	4.34 x10^-9^
**GPCR ligand binding**	36	469	4.43 x10^-9^
**Immune System**	99	2249	4.56 x10^-9^
**Interleukin-1 processing**	6	9	1.50 x10^-7^

Count: enriched protein number in the pathway.

### Protein-protein functional interaction

**Figure 4:**
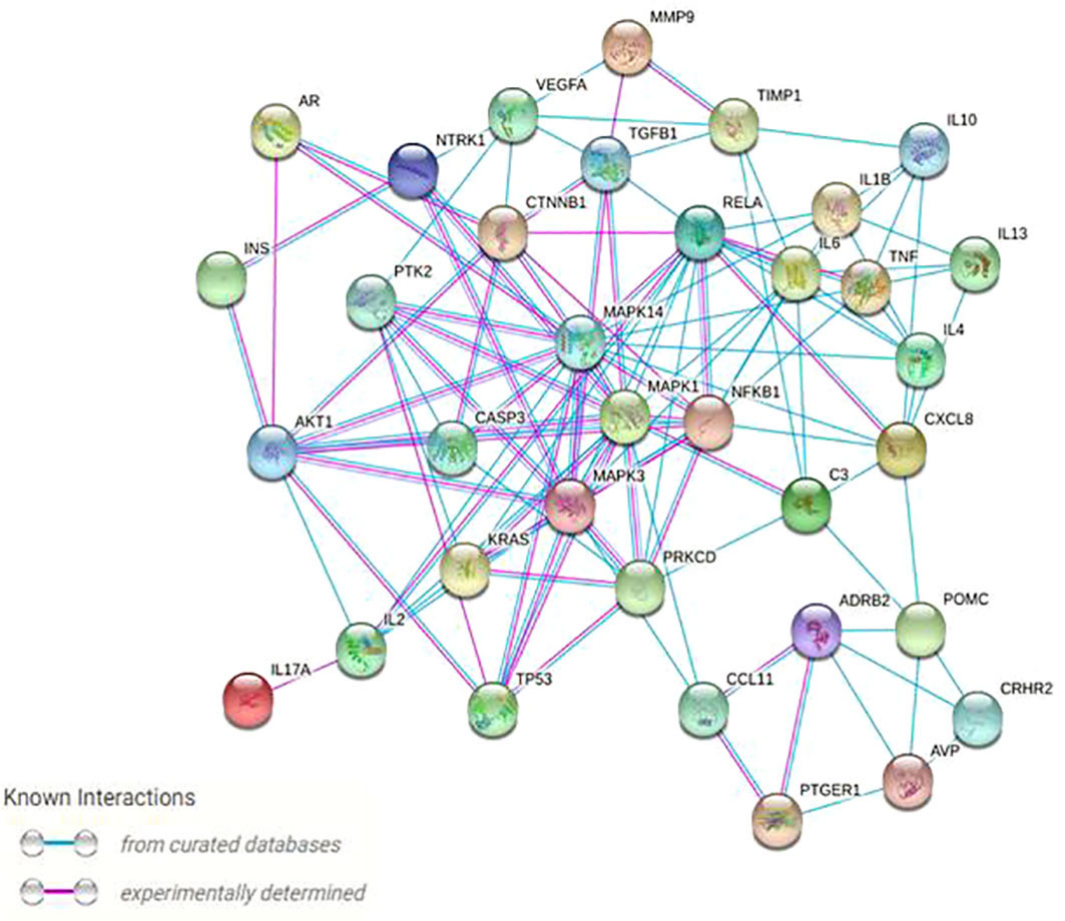
Protein–protein high (confidence score 0.9) physical and functional interactions network of the 35 targeted proteins generated by the String and Centiscape softwares. Network nodes represent proteins; blue edges represent known interactions from curated databases, and pink edges represent experimentally determined interactions.

To be retained, proteins had to exhibit a higher than average degree (>5.27) and betweennes (166.57), to be both first neighbors of the given node and the shortest path linking two nodes. A total of 35 nodes (proteins) with 150 edges (interactions) were: *Mitogen-Activated Protein Kinase 1, 3 and 14; Interleukin 1β, 2, 4, 6, 10, 13 and 17A; C3; Protein Kinase C Delta; Neurotrophic Receptor Tyrosine Kinase 1; Recombinant Insulin; Adrenoceptor Beta 2; Transcription factor p65; Transforming Growth Factor Beta 1; C-X-C Motif Chemokine Ligand 8; Tumor necrosis factor; Nuclear Factor Kappa B Subunit 1; Caspase-3 precursor; Tumor protein 53; Matrix metallopeptidase 9; Vascular endothelial growth factor A; Metallopeptidase Inhibitor 1; Human Corticotrophin Releasing Hormone Receptor 2; Androgen receptor; Prostaglandin E Receptor 1; Arginine Vasopressin; Proopiomelanocortin; KRAS Proto-Oncogene; Protein kinase B; C-C Motif Chemokine Ligand 11; Catenin Beta 1; Protein Tyrosine Kinase 2* (**Table 2**). Finally, prevailing protein-protein interactions network was visualized with STRING (**Figure 4**). These proteins were considered to be the most modulated in the symptomatology of endometriosis and thus could explain the underlying biological mechanisms of endometriosis.

**Table 2:** Proteins with higher than average betweenness and degree in the protein-protein interaction network.

Protein Name	UniProtKB ID	Betweenness (average 166.57)	Degree (average 5.27)
***POMC***	P01189	2562.6	25
***CXCL8***	P10145	1927.9	27
***MAPK1***	P28482	1879.0	28
***AKT1***	P31749	1531.5	21
***CCL11***	P51671	1332.1	8
***IL6***	P05231	1289.2	27
***IL2***	P60568	1275.9	19
***MAPK3***	P27361	1258.2	26
***INS***	P01308	1251.5	11
***VEGFA***	P15692	1183.3	20
***C3***	P01024	1177.1	20
***NFKB1***	P19838	1151.5	21
***MAPK14***	Q16539	875.4	17
***KRAS***	P01116	870.6	16
***CTNNB1***	P35222	853.3	13
***TNF***	P01375	600.7	24
***IL4***	P05112	582.8	17
***PTGER1***	P34995	583.3	8
***IL10***	P22301	533.6	21
***RELA***	Q04206	493.7	21
***IL1B***	P01584	460.8	22
***IL13***	P35225	456.7	16
***NTRK1***	P04629	440.4	8
***TP53***	P04637	425.4	17
***ADRB2***	P07550	420.2	12
***CRHR2***	Q13324	379.1	12
***CASP3***	P42574	374.4	10
***AVP***	P01185	352.2	16
***MMP9***	P14780	345.0	12
***TGFB1***	P01137	282.8	10
***AR***	P10275	282.1	8
***PRKCD***	Q05655	269.8	13
***IL17A***	Q16552	256.8	9
***PTK2***	Q05397	253.7	11
***TIMP1***	P01033	222.1	10

## Discussion

This study provided a text-mining approach that tapped data from bioinformatics banks, with the aim of investigating the protein network related to the symptomatology of endometriosis. A holistic approach was favored to understand the associated complex biological mechanisms behind this symptomatology. Expressed protein may support the advent of some symptoms, which should help health professionals and clinicians in their investigations. As an attempt to address the knowledge gaps surrounding this disease, a special feature of our approach relies on the interest in all the genes collected in the literature as being related to the symptomatology of endometriosis. These genes are often detected based on the results of isolated experimentations.

Thirty-five key proteins were identified in the current study as potential modulators of the symptomatology of endometriosis. Inflammation in endometriosis, widely supported by existing literature, is reflected by an overexpression of inflammatory cytokines, inhibition of endothelial function and hormonal dysregulations (10, 33). A pro-inflammatory cytokine such as *Interleukin-1 β* (*IL-1β*) may enhance the proliferation of endometriotic cells. *IL-1β* can also trigger the production of *IL-6* and *IL-8* (other pro-inflammatory cytokines), which are involved in more proliferation and the decrease of apoptotic rate (14). Inflammation state not only leads to dysmenorrhea, dyspareunia and infertility (34) but also cause oxidative stress connected to poor‐quality embryos and immature oocytes (14). The over-activation of macrophages and mast cells in endometriosis produces *IL-1β*, *TNF-α*, *IL-6*, and *IL-10* (35) found in our analysis. Even if the involvement of the *IL-10* pathway in endometriosis is still poorly understood, the *IL-10 signaling pathway* has an ability to block cytokines and chemokines from macrophages being the root of inflammatory processes (36, 37). Moreover, anti-inflammatory *signaling pathways IL-4 and IL-13* are involved in the cellular immune response, particularly T helper type 2 (38). *IL-4* and *IL-13* type I and II receptor signaling pathways are linked to Signal Transducer and Activator of Transcription 6 (*STAT6*) expression (39), responsible for mediating *IL-4* and *IL-13* immune signaling, increase proliferation, adhesion and viability of endometriosis lesions (38). *Suppressor Of Cytokine Signaling protein 1* (*SOCS1*) inhibits *STAT6* expression and has a protective effect by activating cell apoptosis (39). *SOCS1* dysregulation may exacerbate the *IL-4* and *IL-13* properties associated with endometriosis.

Hence, the findings from the current study pinpoint that both pro-inflammatory and anti-inflammatory pathways are involved in the symptomatology of endometriosis. This was assumed to play a paradoxical role in acute and chronic phases of the disease due to pathological immune imbalance (38). Interestingly, a study on the mouse model has shown that pre-existing peritoneal inflammation did not contribute to the development of endometriosis and could even reduce it (33). This supports the need to provide understanding of the precise role of inflammation in this disease.

Some previously identified targets in the literature (i.e. *IL-1, IL-6, IL-4, and VEGF*) were emphasized by our analysis. The *IL-1 pathway* is a pro-inflammatory cytokine that activated *NF-kB* inflammatory pathways (34). Dysregulation of cytokines and *NF-kB* factor induces both an inflammatory process and an immune system dysfunction involved in endometriosis (40). The *NF-kB* biological pathway has several subunits such as the *p65* involved in the regulation of cell survival. The pathway expressed by *p65* also plays a role in the inflammatory response and contributes to angiogenesis and metastasis survival (41–43). *NF-kB1* subunit promotes the expression of inflammatory cytokines (44, 45).

Similar to the existing literature, the implication of *TNF-α* is again emphasized in this study on **Figure 4**, portraying the most important protein interactions. *TNF-α*, which plays an essential role in increasing proliferative potential, acts primarily as a precursor in initiating an inflammatory response by activating a cascade of other cytokines, such as *IL-1, IL-6* and the vascular endothelial growth factor (*VEGF*) (7, 46). Abnormal *VEGF* levels may impair the process of angiogenesis, which is favorable to embryonic implantation, thus justifying the high miscarriage rate (47, 48). The current study also highlighted some lesser-known targets (i.e. *PGE1, AVP*) (49–51), which may require deeper investigations. Excessive expression of *Prostaglandin E receptor 1* (*PGE1*) is linked to an inflammatory reaction as for tumor protein 53 (*p53*). Unlike our finding, no link between endometriosis and arginine vasopressin (*AVP*) or Caspase-3 has been reported in the literature, and this may require more exploration (46–48, 52). Furthermore, the *KRAS* protein, stressed in the protein network (**Figure 4**) is a factor that is overexpressed in skeletal muscle and myocardium, uterus, adrenal cortex and some bone marrow stem cells (53). A mutation of *KRAS* can cause hyperplasia. This may explain the occurrence of endometrial hyperplasia in endometriosis (49, 54). *TGF-β1* also plays a role in muscle diseases by inhibiting myogenesis and regeneration (55). This involves the development of fibrosis or atrophy of the muscle skeleton (56, 57). Finally, our study pointed out *Neurotrophic Receptor Tyrosine Kinase 1* (*NTRK1*) protein, which may be involved in neuropathic pain. Nerve growth factor (*NGF*) is the main ligand of *NTRK1* (26). It was shown that *NGF* is directly associated with pelvic pain and more specifically with dysmenorrhea, dyspareunia, painful bladder syndrome and irritable bowel syndrome (58, 59). These pains reach the nervous system by provoking a nociception. Such a damage to the nervous system would lead to neuropathic or neuroinflammatory pain (59, 60). In summary, this apparent crosstalk between immune cells, nerves, and central pain pathways is providing an opportunity to develop more targeted therapies against endometriosis and its symptoms (20).

Although the proteins identified should be taken with caution, given the heterogeneity of the studied tissues, the different forms of the disease and techniques used, the current study provides an overall picture of the proteins involved in the symptomatology of endometriosis. In future studies, it would be interesting to examine the involvement of these proteins given the stage of endometriosis and the phases of the menstrual cycle. It is noteworthy that protein interactions that are not already known in the STRING database may lead to discarding key targets involved in the symptomatology of endometriosis. Some proteins have been deeply investigated in the literature while others are rarely studied proteins, and thus may not be detected during text-mining. Also, the network of proteins obtained does not allow weighing to them according to the importance of their involvement in the disease. Thus this approach proves to be complementary to other studies exploring the completeness of the genome (e.g. Genome-wide association studies, GWAS) or using the Gene Expression Omnibus database (GEO) to study differentially expressed genes (61–63). Our study still remains an exploratory analysis that was based on the general symptomatology of endometriosis. We do not get any information on either the different forms of endometriosis or the different stages of the disease. Potentially, this approach can be replicated at a higher level of detail to allow comparing the biological implications of eutopic and ectopic endometrium. In the same way, the query can be refined to select articles from different stages of the disease (early or advanced stage) or from different phases of the menstrual cycle. Hence, the comparison of the signaling pathways related to the early stage of endometriosis (with a search for genes in the articles involved in the early stage of the disease) and the more advanced stage warrants attention. Finally, because Pubtator tools may not have perfect discriminative ability in distinguishing between genes and alleles, some overlapping cannot be completed ruled out.

Taken together, our data highlight that pathways associated to endometriosis symptomatology have sometimes paradoxical roles, certainly resulting in a loss of balance, and these may be time-dependent. Then, developing strategies to enhance their protective effects or to combat their pathological responses at specific stages of the disease could prove therapeutic potential for endometriosis. In conclusion, our study identifies 35 interrelated key proteins with the highest ability to control pathways associated to endometriosis symptomatology. While some proteins such as *IL-1β, IL-6, IL-4, and VEGF* are largely evaluated, our data are suggestive of further investigation on proteins such as *PGE1* and *AVP*. Our study prioritizes potential biomarkers and key targets, and further assessing them in endometriosis could help for the development of diagnostic tools and therapeutic strategies for endometriosis.

## Data availability statement

The original contributions presented in the study are included in the article/**Supplementary Material**. Further inquiries can be directed to the corresponding author.

## Ethics statement

Ethical review and approval was not required for the study on human participants in accordance with the local legislation and institutional requirements. Written informed consent for participation was not required for this study in accordance with the national legislation and the institutional requirements.

## Author contributions

FE and MF designed the study, contributed to the methodology, data curation and execution of the study, analyzed the data and wrote the manuscript. KB and ED-D contributed to the methodology and reviewed the manuscript. AL, ML, and AN reviewed the manuscript. BG and DZ designed the study and revised the manuscript providing their expertise. All authors contributed to the article and approved the submitted version.

## Conflict of interest

The authors declare that the research was conducted in the absence of any commercial or financial relationships that could be construed as a potential conflict of interest.

## Publisher’s note

All claims expressed in this article are solely those of the authors and do not necessarily represent those of their affiliated organizations, or those of the publisher, the editors and the reviewers. Any product that may be evaluated in this article, or claim that may be made by its manufacturer, is not guaranteed or endorsed by the publisher.

## Supplementary material

Supplementary Figure 1 Cumulative number of publications related to endometriosis symptomatology by year (1990 to 2020). Bar chart represents articles meeting the inclusion criteria (i.e human model, women in reproductive age, published in English, which addressed endometriosis and at least one associated clinical sign).

## References

[1] Bulun SE Yilmaz BD Sison C Miyazaki K Bernardi L Liu S. Endometriosis. Endocr Rev (2019) 40(4):1048–79. doi: 10.1210/er.2018-00242 PMC6693056 30994890

[2] Smolarz B Szyłło K Romanowicz H. Endometriosis: Epidemiology, classification, pathogenesis, treatment and genetics (Review of literature). Int J Mol Sci (2021) 22(19):10554. doi: 10.3390/ijms221910554 34638893 PMC8508982

[3] Prodromidou A Machairas N Paspala A Hasemaki N Sotiropoulos GC. Diagnosis, surgical treatment and postoperative outcomes of hepatic endometriosis: A systematic review. Ann Hepatol (2020) 19(1):17–23. doi: 10.1016/j.aohep.2019.08.006 31630985

[4] Zhu J Xue X He Z Zhang J Sun H. Using network pharmacology and molecular docking to explore the underlying anti-inflammatory mechanism of wuyao-danshen to treat endometriosis. Ann Transl Med (2022) 10(4):198. doi: 10.21037/atm-22-419 35280377 PMC8908112

[5] Carpinello OJ Sundheimer LW Alford CE Taylor RN DeCherney AH. Endometriosis. In: Feingold KR Anawalt B Boyce A Chrousos G de Herder WW Dungan K, editors. Endotext. South Dartmouth, MA: MDText.com, Inc (2000).

[6] Chapron C Marcellin L Borghese B Santulli P. Rethinking mechanisms, diagnosis and management of endometriosis. Nat Rev Endocrinol (2019) 15(11):666–82. doi: 10.1038/s41574-019-0245-z 31488888

[7] Koninckx PR Ussia A Adamyan L Tahlak M Keckstein J Wattiez A. The epidemiology of endometriosis is poorly known as the pathophysiology and diagnosis are unclear. Best Pract Res Clin Obstet Gynaecol (2021) 71:14–26. doi: 10.1016/j.bpobgyn.2020.08.005 32978068

[8] Klemmt PAB Starzinski-Powitz A. Molecular and cellular pathogenesis of endometriosis. Curr Womens Health Rev (2018) 14(2):106–16. doi: 10.2174/1573404813666170306163448 PMC5925869 29861704

[9] Coxon L Horne AW Vincent K. Pathophysiology of endometriosis-associated pain: A review of pelvic and central nervous system mechanisms. Best Pract Res Clin Obstet Gynaecol (2018) 51:53–67. doi: 10.1016/j.bpobgyn.2018.01.014 29525437

[10] García-Gómez E Vázquez-Martínez ER Reyes-Mayoral C Cruz-Orozco OP Camacho-Arroyo I Cerbón M. Regulation of inflammation pathways and inflammasome by sex steroid hormones in endometriosis. Front Endocrinol (2020) 10:935. doi: 10.3389/fendo.2019.00935 PMC7000463 32063886

[11] Soliman AM Coyne KS Gries KS Castelli-Haley J Snabes MC Surrey ES. The effect of endometriosis symptoms on absenteeism and presenteeism in the workplace and at home. J Manag Care Spec Pharm (2017) 23(7):745–54. doi: 10.18553/jmcp.2017.23.7.745 PMC10398072 28650252

[12] Fiala L Lenz J Bob P. Effect of psychosocial trauma and stress on sexual dysfunction in women with endometriosis. Med (Baltimore) (2021) 100(31):e26836. doi: 10.1097/MD.0000000000026836 PMC8341311 34397850

[13] Burney RO Giudice LC. Pathogenesis and pathophysiology of endometriosis. Fertil Steril (2012) 98(3):511–9. doi: 10.1016/j.fertnstert.2012.06.029 PMC3836682 22819144

[14] Samimi M Pourhanifeh MH Mehdizadehkashi A Eftekhar T Asemi Z. The role of inflammation, oxidative stress, angiogenesis, and apoptosis in the pathophysiology of endometriosis: Basic science and new insights based on gene expression. J Cell Physiol (2019) 234(11):19384–92. doi: 10.1002/jcp.28666 31004368

[15] Vercellini P Viganò P Somigliana E Fedele L. Endometriosis: pathogenesis and treatment. Nat Rev Endocrinol (2014) 10(5):261–75. doi: 10.1038/nrendo.2013.255 24366116

[16] Heydari S Kashani L Noruzinia M. Dysregulation of angiogenesis and inflammatory genes in endometrial mesenchymal stem cells and their contribution to endometriosis. Iran J Allergy Asthma Immunol (2021) 20(6):740. doi: 10.18502/ijaai.v20i6.8025 34920657

[17] Zarezadeh Mehrabadi A Aghamohamadi N Khoshmirsafa M Aghamajidi A Pilehforoshha M Massoumi R. The roles of interleukin-1 receptor accessory protein in certain inflammatory conditions. Immunology (2022) 166(1):38–46. doi: 10.1111/imm.13462 35231129

[18] Zhang T De Carolis C Man GCW Wang CC. The link between immunity, autoimmunity and endometriosis: a literature update. Autoimmun Rev (2018) 17(10):945–55. doi: 10.1016/j.autrev.2018.03.017 30107265

[19] Méar L Com E Fathallah K Guillot L Lavigne R Guével B. The eutopic endometrium proteome in endometriosis reveals candidate markers and molecular mechanisms of physiopathology. Diagn (Basel) (2022) 12(2):419. doi: 10.3390/diagnostics12020419 PMC8870972 35204508

[20] Saunders PTK Horne AW. Endometriosis: Etiology, pathobiology, and therapeutic prospects. Cell (2021) 184(11):2807–24. doi: 10.1016/j.cell.2021.04.041 34048704

[21] Chen S Chai X Wu X. Bioinformatical analysis of the key differentially expressed genes and associations with immune cell infiltration in development of endometriosis. BMC Genom Data (2022) 23:20. doi: 10.1186/s12863-022-01036-y 35303800 PMC8932180

[22] Peng Y Peng C Fang Z Chen G. Bioinformatics analysis identifies molecular markers regulating development and progression of endometriosis and potential therapeutic drugs. Front Genet (2021) 12:622683. doi: 10.3389/fgene.2021.622683 34421979 PMC8372410

[23] Wei C-H Allot A Leaman R Lu Z. PubTator central: automated concept annotation for biomedical full text articles. Nucleic Acids Res (2019) 47(W1):W587–W93. doi: 10.1093/nar/gkz389 PMC6602571 31114887

[24] PubTator. NLM/NCBI BioNLP research group. discover biomedical entities in more than 30 million biomedical publications (2013). Available at: https://www.ncbi.nlm.nih.gov/research/pubtator/.

[25] Wei C-H Kao H-Y Lu Z. GNormPlus: An integrative approach for tagging genes, gene families, and protein domains. BioMed Res Int (2015) 2015:918710. doi: 10.1155/2015/918710 26380306 PMC4561873

[26] UniProt. The UniProt consortium. UniProt: the universal protein knowledgebase. Nucleic Acids Res (2017) 45(D1):D158–69. doi: 10.1093/nar/gkw1099 PMC5210571 27899622

[27] UniProt. (2002). Available at: https://www.uniprot.org/.

[28] García-Moreno A López-Domínguez R Ramirez-Mena A Pascual-Montano A Aparicio-Puerta E Hackenberg M. GeneCodis 4: Expanding the modular enrichment analysis to regulatory elements. bioRXiv (2021), 1–9. doi: 10.1101/2021.04.15.439962 PMC8945021 35327392

[29] GeneCodis. Gene annotations co-occurence discovery (2007). Available at: https://genecodis.genyo.es/.

[30] Fabregat A Jupe S Matthews L Sidiropoulos K Gillespie M Garapati P. The reactome pathway knowledgebase. Nucleic Acids Res (2018) 46(D1):D649–D55. doi: 10.1093/nar/gkx1132 PMC5753187 29145629

[31] reactome. Find reactions, proteins and pathways (2003). Available at: https://reactome.org/.

[32] Doncheva NT Morris JH Gorodkin J Jensen LJ. Cytoscape StringApp: Network analysis and visualization of proteomics data. J Proteome Res (2019) 18(2):623–32. doi: 10.1021/acs.jproteome.8b00702 PMC6800166 30450911

[33] Jiang L Yan Y Liu Z Wang Y. Inflammation and endometriosis. Front Biosci (Landmark Ed) (2016) 21(5):941–8. doi: 10.2741/4431 27100482

[34] Malvezzi H Hernandes C Piccinato CA Podgaec S. Interleukin in endometriosis-associated infertility-pelvic pain: systematic review and meta-analysis. Reproduction (2019) 158(1):1–12. doi: 10.1530/REP-18-0618 30933927

[35] Wei Y Liang Y Lin H Dai Y Yao SJ. Autonomic nervous system and inflammation interaction in endometriosis-associated pain. Neuroinflammation (2020) 17(1):80. doi: 10.1186/s12974-020-01752-1 PMC7060607 32145751

[36] Moore KW de Waal Malefyt R Coffman RL O’Garra A. Interleukin-10 and the interleukin-10 receptor. Annu Rev Immunol (2001) 19:683–765. doi: 10.1146/annurev.immunol.19.1.683 11244051

[37] Hogg C Panir K Dhami P Rosser M Mack M Soong D. Macrophages inhibit and enhance endometriosis depending on their origin. Proc Natl Acad Sci U S A (2021) 118(6):e2013776118. doi: 10.1073/pnas.2013776118 33536334 PMC8017702

[38] Zhou W-J Yang H-L Shao J Mei J Chang K-K Zhu R. Anti-inflammatory cytokines in endometriosis. Cell Mol Life Sci (2019) 76(11):2111–32. doi: 10.1007/s00018-019-03056-x PMC11105498 30826860

[39] Hershey GKK. IL-13 receptors and signaling pathways: An evolving web. J Allergy Clin Immunol (2003) 111(4):677–90. doi: 10.1067/mai.2003.1333 12704343

[40] Adewuyi EO Sapkota Y Auta A Yoshihara K Nyegaard M Griffiths LR. Shared molecular genetic mechanisms underlie endometriosis and migraine comorbidity. Genes (Basel) (2020) 11(3):268. doi: 10.3390/genes11030268 PMC7140889 32121467

[41] Bozkurt M Şahin L Ulaş M. Hysteroscopic polypectomy decreases NF-κB1 expression in the mid-secretory endometrium of women with endometrial polyp. Eur J Obstet Gynecol Reprod Biol (2015) 189:96–100. doi: 10.1016/j.ejogrb.2015.03.032 25898371

[42] Kim SH Ihm HJ Oh YS Chae HD Kim CH Kang BM. Increased nuclear expression of nuclear factor kappa-b p65 subunit in the eutopic endometrium and ovarian endometrioma of women with advanced stage endometriosis. Am J Reprod Immunol (2013) 70(6):497–508. doi: 10.1111/aji.12161 24118362

[43] Wang J Zhou M Zhang Q-G Xu J Lin T Zhou R-F. Prognostic value of expression of nuclear factor kappa-B/p65 in non-GCB DLBCL patients. Oncotarget (2016) 8(6):9708−16. doi: 10.18632/oncotarget.14182 PMC5354765 28039454

[44] Lorenzini T Fliegauf M Klammer N Frede N Proietti M Bulashevska A. Characterization of the clinical and immunologic phenotype and management of 157 individuals with 56 distinct heterozygous NFKB1 mutations. J Allergy Clin Immunol (2020) 46(4):901–11. doi: 10.1016/j.jaci.2019.11.051 PMC8246418 32278790

[45] Wang Y Wu B Zhang M Miao H Sun J. Significant association between rs28362491 polymorphism in NF-κB1 gene and coronary artery disease: a meta-analysis. BMC Cardiovasc Disord (2020) 20(1):278. doi: 10.1186/s12872-020-01568-0 32513188 PMC7282174

[46] Sarker A Ginn R Nikfarjam A O'Connor K Smith K Jayaraman S. Utilizing social media data for pharmacovigilance: A review. J BioMed Inform (2015) 54:202–12. doi: 10.1016/j.jbi.2015.02.004 PMC4408239 25720841

[47] Chen H Deng X Yang Y Shen Y Chao L Wen Y. Expression of GRIM-19 in missed abortion and possible pathogenesis. Fertil Steril (2015) 103(1):138–46.e3. doi: 10.1016/j.fertnstert.2014.10.012 25455534

[48] Harmsen MJ Wong CFC Mijatovic V Griffioen AW Groenman F Hehenkamp WJK. Role of angiogenesis in adenomyosis-associated abnormal uterine bleeding and subfertility: a systematic review. Hum Reprod Update (2019) 25(5):647–71. doi: 10.1093/humupd/dmz024 PMC6737562 31504506

[49] Bouaziz J Mashiach R Cohen S Kedem A Baron A Zajicek M. How artificial intelligence can improve our understanding of the genes associated with endometriosis: Natural language processing of the PubMed database. BioMed Res Int (2018) 2018:1–7. doi: 10.1155/2018/6217812 PMC5884286 29750165

[50] Liu F Lv X Yu H Xu P Ma R Zou K. In search of key genes associated with endometriosis using bioinformatics approach. Eur J Obstet Gynecol Reprod Biol (2015) 194:119–24. doi: 10.1016/j.ejogrb.2015.08.028 26366788

[51] Liu J-L Zhao M. Prioritization of susceptibility genes for ectopic pregnancy by gene network analysis. Int J Mol Sci (2016) 17(2):191. doi: 10.3390/ijms17020191 PMC4783925 26840308

[52] Sarkar S Hobson AR Hughes A Growcott J Woolf CJ Thompson DG. The prostaglandin E2 receptor-1 (EP-1) mediates acid-induced visceral pain hypersensitivity in humans. Gastroenterology (2003) 124(1):18–25. doi: 10.1053/gast.2003.50022 12512025

[53] Timar J Kashofer K. Molecular epidemiology and diagnostics of KRAS mutations in human cancer. Cancer Metastasis Rev (2020) 39(4):1029–38. doi: 10.1007/s10555-020-09915-5 PMC7680318 32725342

[54] Sideris M Emin EI Abdullah Z Hanrahan J Stefatou KM Sevas V. The role of KRAS in endometrial cancer: A mini-review. Anticancer Res (2019) 39(2):533–9. doi: 10.21873/anticanres.13145 30711927

[55] Yang Y Zhang N Lan F Van Crombruggen K Fang L Hu G. Transforming growth factor-beta 1 pathways in inflammatory airway diseases. Allergy (2014) 69(6):699–707. doi: 10.1111/all.12403 24750111

[56] Abrigo J Simon F Cabrera D Cordova G Trollet C Cabello-Verrugio C. Central role of transforming growth factor type beta 1 in skeletal muscle dysfunctions: An update on therapeutic strategies. Curr Protein Pept Sci (2018) 19(12):1189–200. doi: 10.2174/1389203718666171117101916 29150918

[57] Loverro G Maiorano E Napoli A Selvaggi L Marra E Perlino E. Transforming growth factor-beta 1 and insulin-like growth factor-1 expression in ovarian endometriotic cysts: a preliminary study. Int J Mol Med (2001) 7(4):423–9. doi: 10.3892/ijmm.7.4.423 11254886

[58] Kajitani T Maruyama T Asada H Uchida H Oda H Uchida S. Possible involvement of nerve growth factor in dysmenorrhea and dyspareunia associated with endometriosis. Endocr J (2013) 60(10):1155–64. doi: 10.1507/endocrj.EJ13-0027 23883529

[59] Zheng P Zhang W Leng J Lang J. Research on central sensitization of endometriosis-associated pain: a systematic review of the literature. J Pain Res (2019) 12:1447–56. doi: 10.2147/JPR.S197667 PMC6514255 31190954

[60] Orr NL Wahl KJ Lisonek M Joannou A Noga H Albert A. Central sensitization inventory in endometriosis. Pain (2022) 163(2):e234–e45. doi: 10.1097/j.pain.0000000000002351 34030173

[61] Nyholt DR Low S-K Anderson CA Painter JN Uno S Morris AP. Genome-wide association meta-analysis identifies new endometriosis risk loci. Nat Genet (2012) 44(12):1355–9. doi: 10.1038/ng.2445 PMC3527416 23104006

[62] Rahman MH Peng S Hu X Chen C Rahman MR Uddin S. A network-based bioinformatics approach to identify molecular biomarkers for type 2 diabetes that are linked to the progression of neurological diseases. Int J Environ Res Public Health (2020) 17(3):1035. doi: 10.3390/ijerph17031035 PMC7037290 32041280

[63] Rahman MH Rana HK Peng S Hu X Chen C Quinn JMW. Bioinformatics and machine learning methodologies to identify the effects of central nervous system disorders on glioblastoma progression. Brief Bioinform (2021) 22(5):bbaa365. doi: 10.1093/bib/bbaa365 33406529

