# Supplementary figures and images for: Exploration of the core protein network under endometriosis symptomatology using a computational approach
Source: Front Endocrinol (Lausanne). 2022 Sep 2;13:869053. doi: 10.3389/fendo.2022.869053 (PMC9478376; doi:10.3389/fendo.2022.869053)

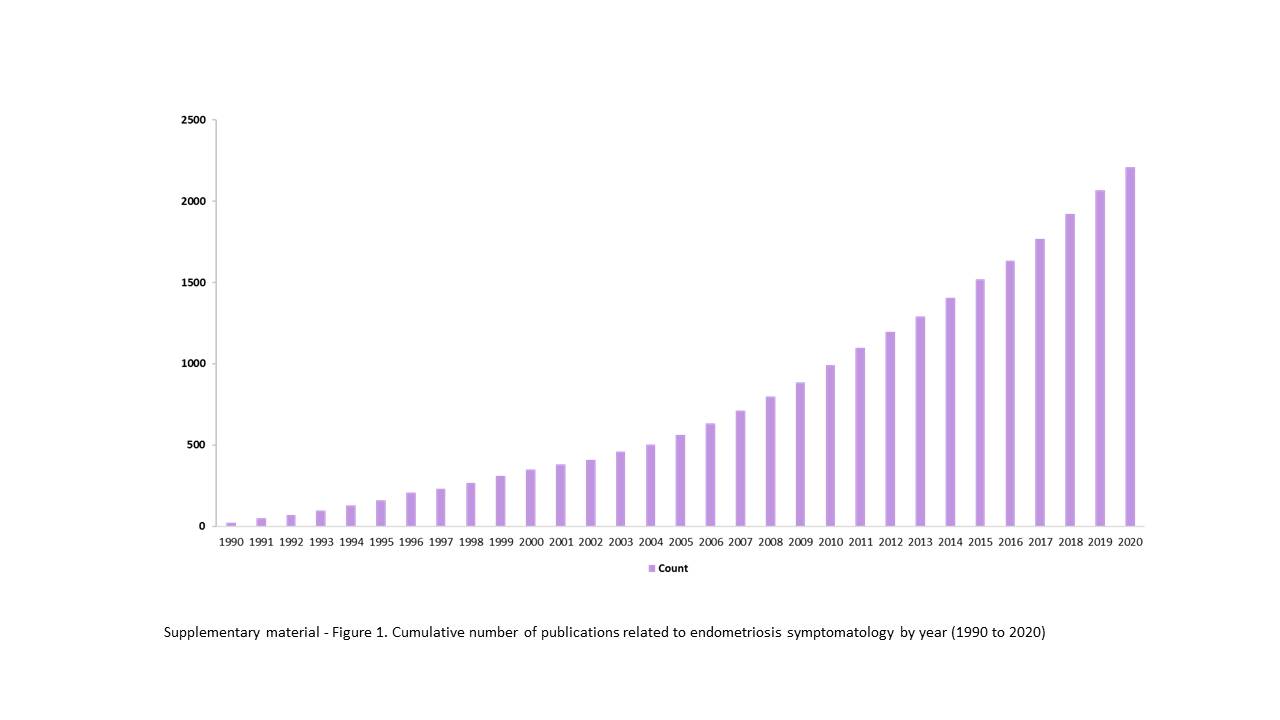

Supplement: Supplementary Figure 1 — Cumulative number of publications related to endometriosis symptomatology by year (1990 to 2020). Bar chart represents articles meeting the inclusion criteria (i.e human model, women in reproductive age, published in English, which addressed endometriosis and at least one associated clinical sign). [file Image_1.jpeg]
